# Cystinuria: an inborn cause of urolithiasis

**DOI:** 10.1186/1750-1172-7-19

**Published:** 2012-04-05

**Authors:** Thomas Eggermann, Andreas Venghaus, Klaus Zerres

**Affiliations:** 1 Institute of Human Genetics, University Hospital, RWTH Aachen, Pauwelsstr. 30, Aachen, D-52074, Germany

**Keywords:** Cystinuria, *SLC3A1*, *SLC7A9*, Urolithiasis, Genetic testing

## Abstract

Cystinuria (OMIM 220100) is an inborn congenital disorder characterised by a defective cystine metabolism resulting in the formation of cystine stones. Among the heterogeneous group of kidney stone diseases, cystinuria is the only disorder which is exclusively caused by gene mutations. So far, two genes responsible for cystinuria have been identified: *SLC3A1* (chromosome 2p21) encodes the heavy subunit rBAT of a renal b^0,+^ transporter while *SLC7A9* (chromosome 19q12) encodes its interacting light subunit b^0,+^AT. Mutations in *SLC3A1* are generally associated with an autosomal-recessive mode of inheritance whereas *SLC7A9* variants result in a broad clinical variability even within the same family. The detection rate for mutations in these genes is larger than 85%, but it is influenced by the ethnic origin of a patient and the pathophysiological significance of the mutations. In addition to isolated cystinuria, patients suffering from the hypotonia-cystinuria syndrome have been reported carrying deletions including at least the *SLC3A1* and the *PREPL* genes in 2p21. By extensive molecular screening studies in large cohort of patients a broad spectrum of mutations could be identified, several of these variants were functionally analysed and thereby allowed insights in the pathology of the disease as well as in the renal trafficking of cystine and the dibasic amino acids. In our review we will summarize the current knowledge on the physiological and the genetic basis of cystinuria as an inborn cause of kidney stones, and the application of this knowledge in genetic testing strategies.

## Review

The incidence of kidney stone diseases has increased over the last decades in the industrial countries to nearly 1.5% in 2000, about 5% of all women and 12% of men will develop a kidney stone once in their life [1]. The incidence in childhood is lower and has been estimated to be approximately 0.15% (for review: [2]). Interestingly, about 40% of children with kidney stones have a positive familial history whereas urolithiasis in adults often occurs sporadically.

In case of stone formation, the qualitative analysis is one of the most important diagnostic measures: calcium and oxalate are the main stone components in the European population detectable in more than 75% of patients, whereas phosphate, cystine, purine and other stones are often rare (for review: [2]). Many lithogenic and inhibitory factors are involved in the aetiology of stone formation which is significantly influenced by fluid intake and dietary factors.

Indeed, a genetic predisposition can be observed in many disorders associated with kidney stones, but cystinuria is the only entity solely caused by genetic mutations. Among adults cystine stones account for only 1-2% of all urinary nephrolithiasis patients, but in children 6-8% of patients suffer from cystine stones [3].

Cystinuria (OMIM 220100) is characterised by the defective reabsorption of cystine, lysine, ornithine and arginine in the brush border membrane of the proximal renal tubule (S3 segment) and in the epithelial cells of the gastrointestinal tract (for review: [4]). Although all four amino acids reach high urinary concentrations, only the resulting urinary hyperexcretion of cystine leads to precipitation in the distal tubule and formation of cystine stones due to its low solubility of at low pH. In patients with large genomic deletions affecting the *SLC3A1* gene as well as at least the neighboured *PREPL* gene in 2p21, urolithiasis is additionally associated with hypotonia and further clinical symptoms (see below).

## Clinical findings and classification

The first diagnosis of cystinuria is commonly based on the finding of cystine stones which typically show characteristic cystine crystals. The crystals are usually hexagonal, translucent and white. Upon removal, the stones may be pink or yellow in color, but later they turn to greenish due to exposure to air. Cystine crystals are visible in 17 to 25% of urine samples of patients with cystinuria (for review: [2]). The stones may be identified by a positive nitroprusside cyanide test. Most stones are exceptionally formed of cystine, but mixed composition can be observed. The diagnosis can be confirmed by determination of the urinary amino acid excretion: in case of cystinuria urinary cystine excretion is typically increased to >1000 μmoL/g creatinine [5]. However, due to the incomplete expression of the renal amino acid transporters a so-called *transient neonatal cystinuria* can be observed in cystinuria heterozygotes, thus diagnosis before the 4 year of life should be made with caution. [6].

Due to their composition, fragmentation of cystine stones by extracoporal lithothripsy is often difficult, and larger stones generally require percutaneous nephrostomy placement and removed. Episodic stone symptoms make repeated removements of the stones necessary. Indeed, the disorder cause serious damage to the kidneys and surrounding organs, and in some rare cases death if not treated properly. Current treatment of cystinolithiasis is currently focused on prevention of stone formation by reducing cystine excretion and concentration and by reducing cystine to the more soluble cysteine (for review: [4]). However, causative therapies have not yet been developed despite the growing knowledge on the physiological aetiology of the disease.

For clinical purposes, the classification of cystinuria is based on the urinary phenotype of obligate heterozygotes (i.e. parents of patients with a classical course of cystinuria) and three types of cystinuria have been distinguished [7,8]. Whereas type I heterozygotes excrete cystine at normal levels, type II and III heterozygotes show a highly or moderately elevated excretion. However, after the identification of the genetic mutations predisposing to cystinuria, a correlation between the extent of hyperaminoaciduria and the mutation in type II/III heterozygotes could not be established. Therefore both types II and III were summarized as non-type I [9]. Non-type I heterozygotes show a variable urinary hyperexcretion of cystine and the dibasic amino acids, in some carriers stone formation has been reported [10]. As a result, non-type I cystinuria can be regarded as an autosomal-dominant disorder with incomplete penetrance for cystine lithiasis, whereas type I cystinuria mainly follow an autosomal-recessive trait. In addition, patients with a mixed cystinuria have been reported, carrying both type I and non-type I alleles (for review: [4]).

In the majority of cystinuria patients, stone formation occurs within the first two decades of life [11] but a broad intrafamilial variation of the disease has been reported. Males are affected more frequently and severely than females, and males have a larger number of stones. More than 80% of patients develop their first stones within the first two decades, but stones may be formed at any age. In male patients an earlier stone formation can be observed in comparison to females. Around 6% of patients do not form stones [12].

Goodyer et al. [5] observed a preponderance of an early manifestation of stone formation in type I-homozygous patients, whereas non-type I homozygous as well as mixed type patients developed stones later in life. However, this correlation could not be confirmed in molecularly proven patients [13].

## Frequency

Cystinuria is a global disorder with population-specific prevalences, its overall prevalence has been estimated as 1:7000 in neonates [14]. It varies between different populations: the highest frequency has been observed among Libyan Jews with a rate of 1:2,500, in Americans the rate is 1:15,000, and in Sweden 1:100,000 [14,15]. In specific populations it can therefore be regarded as one of the most common autosomal-recessive disorders comparable only to cystic fibrosis. However, due to this limited information about the prevalence of cystinuria in the general population and the broad spectrum of mutations, the frequency of cystinuria carriers can only be estimated. The highest carrier rate of 1:25 in Libyan Jews has been identified by Sidi et al. [16]. In the Swedish population, p.Met467Thr occurs in 0.5% of the general population [17].

## The *SLC3A1* and *SLC7A9* genes and the amino acid transporter system b^0,+^

The involvement of the *SLC3A1* (OMIM 104614) gene in the aetiology of cystinuria could be established in 1994 by establishing linkage of the disease to 2p and by identifying the first mutations in cystinuria patients [18,19]. Further genetic linkage studies indicated that not all cystinuria families were caused by *SLC3A1* defects, and the second cystinuria gene could be localised to chromosome 19q13 [20,21]. In 1999, first mutations in the *SLC7A9* (OMIM 604144) gene were reported [9].

The *SLC3A1* gene spans ~46 kb and includes 10 coding exons [22,23]. The longest transcript (ENST00000260649) is 2989 bp long, the resulting protein rBAT (ENSP00000260649) consists of 685 amino acids. The *SLC7A9* gene comprises 12 exons, 11 of which are coding. The transcript (ENST00000023064) has 1772 bp, the protein b^0,+^AT consists of 487 amino acids.

**Figure 1:**
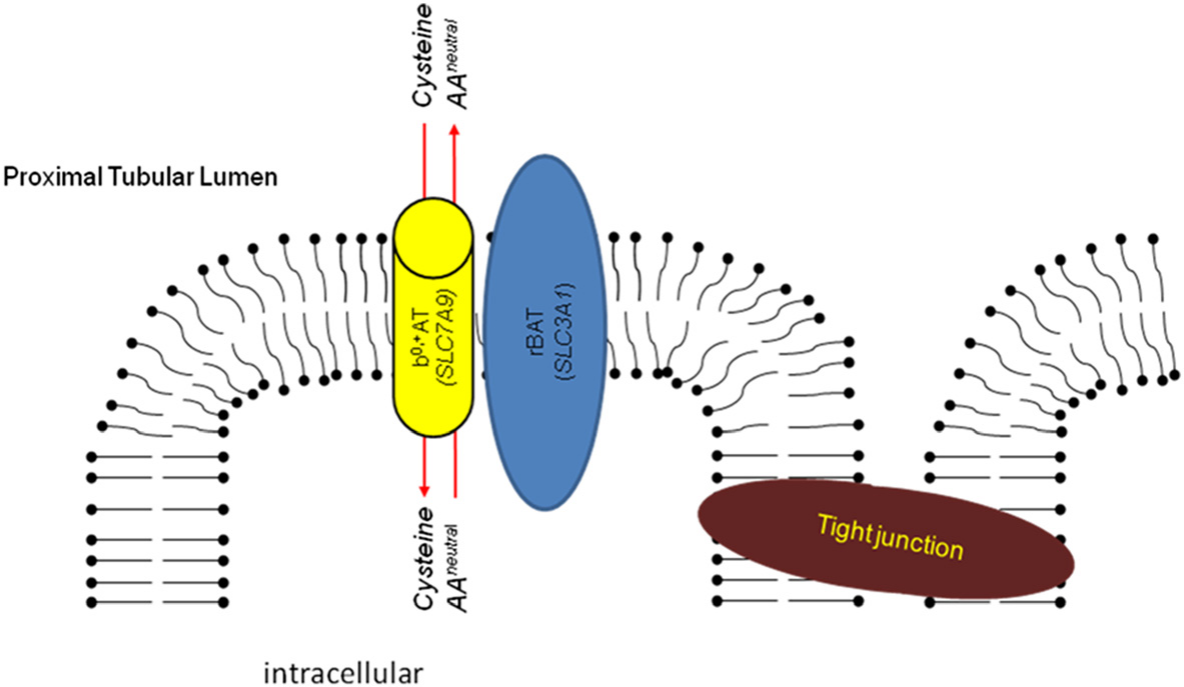
**Cellular localisation and function of rBAT (*SLC3A1*) and b**^0,+^**AT (*SLC7A9*).**

The two genes encode the two subunits of the renal heteromeric amino acid transporter b^0,+^: the heavy subunit rBAT (*SLC3A1*) and the light subunit b^0,+^AT (*SLC7A9*)) (Figure 1)(for review: [4]), both linked by a conserved disulfide bridge. The heavy subunit mediates the localisation of the holotransporter to the plasma membrane whereas the light subunit comprises the catalytic component of the transporter.

The rBAT/b^0,+^ amino acid transporter is expressed in the apical membrane of the epithelial cells of the S1-S2 segments of the proximal tubule and of the small intestine. Via the heterodimeric transporter the dibasic amino acids and cystine are exchanged for other neutral amino acids in a 1:1 stochiometry. rBAT/b^0,+^AT represents the main mechanism of tubular cystine transport and accounts for >90% of renal cystine absorption.

As a result of its biological functions, mutations in *SLC3A1*/rBAT have strong trafficking effects, either by a deficient to assemble with b^0,+^AT or by failure to oligomerize with subsequent degradation(for review: [4]). b^0,+^AT mutations cause a loss of function of the transporter system by defective folding, trafficking, heterodimerization, transport activity or substrate recognition (for review: [4]).

## Genetics and spectrum of mutations

As aforementioned, the classification of cystinuria was historically based on the urinary excretion pattern of heterozygotes. After identification of the molecular basis of the disease a new classification was suggested: the autosomal recessively inherited type I cystinuria which is mainly caused by *SLC3A1* mutations, and the incomplete autosomal dominant non-type I cystinuria associated with *SLC7A9* variants. However, the identification of both *SLC3A1* mutations causing non-type I cystinuria and recessive *SLC7A9* mutations lead to a new strictly molecular classification [11] which does not include the biochemical phenotype: type A cystinuria represents *SLC3A1* mutations, and type B cystinuria includes *SLC7A9* mutations. Thus, three genotypes can be delineated: AA, BB, and the mixed cystinuria AB.

In large screening studies, more than 130 pathogenic variants in *SLC3A1* and nearly 100 mutations in *SLC7A9* have been reported (for review: [4,24]). The observed variants cover the whole spectrum of mutations, ranging from nonsense, missense, splicing, and frameshift mutations to whole and multi-exon imbalances. The significant role of large genomic rearrangements became obvious with the identification of the first deletions/duplications in *SLC3A1*[25] and the characterisation of the relatively frequent duplication affecting exons 5 to 9 (dupE5E9; c.891 + 1524_1618-1600dup) in *SLC3A1*[4,24] accounting for at least 11% of mutated *SLC3A1* alleles (Table 1). A systematic screen for whole exon/multi exon imbalances was recently reported by Bisceglia et al. [24] applying MLPA (multiplex ligation probe-dependend amplification): they could demonstrate that large rearrangements significantly contribute to the spectrum of mutations in both cystinuria genes in Italy.

**Table 1:** Overview on the most frequent cystinuria mutations in a) *SLC3A1*, and b*) SLC7A9* in different ethnic groups

a) *SLC3A1*																			
**Cohort**		**Cohort size(n)°**	p.Tyr151Cys/Asn*	**p.Thr216Met**		p.Arg270X/Leu*	p.Arg362Cys/His*	**p.Arg365Gln/** Trp/Leu/Pro*		p.Arg452Trp/X*		**p.Met467Thr**	**p.Met467Lys**		p.Ser547Trp/Lys*	**dupE5E9**	**Identified Alleles**	**Methods**	**References**
*Exon*			*2*	*3*		*4*	*6*	*6*		*8*		*8*	*8*		*10*	*5-9*			
*Europe*																			
Czech/Slovakia		24	0	5		0	0	1		3		**12**	0		1	ND	29	sequencing	[26]
Germany		75	0	**7**		1	1	5		1		**19**	0		2	13	69	sequencing, qPCR	[25,27-30]
Greece		20	0	**9**		2	0	1		0		**4**	0		0	ND	21	SSCP, RFLP	[31]
		4	0	**8**		0	0	0		0		**0**	0		0	ND	8	sequencing	[25,27,28,30,32]
Italy		168	2	**10**		1	5	5		4		**34**	7		9	11	145	sequencing, MLPA	[24]
		12	0	1		0	0	1		0		**1**	2		0	0	5	sequencing, qPCR	[25,27,28,30,32]
Poland		9	0	1		0	0	0		1		**1**	0		0	2	5	sequencing, qPCR	[29]
Portugal		12	0	0		0	0	0		0		**2**	0		0	4	13	sequencing, MLPA, RNA	[33]
Sweden		43	**8**	0		0	1	0		0		**43**	0		1	ND	53	SSCP	[34]
Spain		142	0	2		0	1	2		2		**12**	0		0	0	63	sequencing, MLPA	[35], for review: [4]
former Yugoslavia		13	0	**15**		0	0	2		0		**2**	0		0	0	8	sequencing, qPCR	[25,27,28,30,32]
*Asia*																			
China		8	0	0		0	0	2		0		**0**	0		1	ND	6	sequencing	[37]
Israel		5	0	0		**10**	0	0		0		**0**	0		0	ND	10	cDNA sequencing	[18]
Japan		36	0	0		0	0	0		0		**0**	0		0	ND	8	RNA SSCP	[38]
Turkey		24	ND	ND		ND	ND	ND		ND		**4**	2		ND	ND	6	RFLP	[39]
Turkey		17	0	**6**		0	0	0		0		**1**	0			0	9	seqencing, qPCR	[25,27,28,30,32]
*North America*																			
Canada (French)		20	0	2		1	0	0		0		**2**	0		0	ND	16	RNA Mismatch	[40]
USA (Texas)		33	0	0		4	0	0		1		**12**	0		0	ND	34	SSCP RNA MistMatch	[41]
**Total**			10	**66**		19	8	19		12		**149**	11		14	30	508		
**Ratio of known alleles (%)**			1.9	**13.0**		3.7	1.6	3.7		2.4		**29.3**	2.2		2.6	**11.1**			
**b**) *SLC7A9*																			
**Cohort**	**Cohort size** **(n)°**		**p.Gly105Arg**	**p.Thr123Met**	**p.Val170Met**		**p.Ala182Thr**	**p.Glu244del**	**p.Ala331Val**		**p.Arg333Trp/Gln**			**p.Pro482Leu**	**c.614dupA**	**Identified Alleles**	**Methods**	**References**	
*Exon*			*4*	*4*	*5*		*5*	*7*	*10*		*10*			*13*	*6*				
*Europe*																			
Czech/Slovakia	24		**3**	2	0		0	0	0		0			0	1	11	sequencing	[26]	
Germany	75		**10**	0	0		2	1	1		3			0	1	32	sequencing, qPCR		
Greece	20		**11**	0	0		0	0	0		4			0	0	22	SSCP, RFLP	[31]	
	4		**0**	0	0		0	0	0		0			0	0	0	sequencing	[25,27,28,30,32]	
Italy	168		**44**	1	0		7	4	0		18			1	2	163	sequencing, MLPA	[24]	
	12		**2**	0	0		0	1	0		1			0	0	6	sequencing, qPCR	[25,27,28,30,32]	
Poland	9		**0**	0	0		0	0	0		0			0	0	1	sequencing, qPCR	[29]	
Portugal	12		**0**	0	0		0	0	0		0			0	1	8	sequencing, MLPA, RNA	[33]	
Sweden	15		**0**	0	0		1	0	0		0			0	0	1	SSCP	[17]	
Spain	142		**14**	9	0		4	1	0		20			0	21	72	sequencing,, MLPA, SSCP	[for review: 4]	
former Yugoslavia	13		**1**	0	0		0	0	0		0			0	0	3	sequencing, qPCR	[25,27,28,30,32]	
*Asia*																			
China	8		**0**	0	0		0	0	0		0			0	0	7	sequencing	[35]	
Israel, Libyan	8		**ND**	ND	16		ND	ND	ND		ND			ND	ND	16	cDNA sequencing	[16]	
Japan	39		**0**	0	0		0	0	0		3			**56**		66	RNA SSCP	[42]	
Turkey	17		**1**	0	0		0	0	4		0			0	0	12	seqencing, qPCR	[25,27,28,30,32]	
	13		**0**	0	0		0	0	0		0			0	1	5	SSCP	[35]	
*North America* Canada (French)																			
**Total**			**86**	12	16		14	7	5		49			57	27	425			
													
**Ratio of known alleles**			**20.2**	2.8	3.8		3.3	1.6	1.2		11.5			13.4	6.4				

(frequent mutations or population specific frequencies are printed in bold face. ° total number of patients screened in this study, including classified and unclassified patients * different basepair substitutions have been reported affecting the same codon. # only single study cohorts were screened for this variant, here the total number of alleles is 271; RFLP restriction fragment length polymorphism; qPCR quantitative PCR, SSCP single strand conformation polymorphism analysis, ND not determined).

In single patients, three pathogenic mutations can be detected [27,31]. Indeed, the identification of three recessive variants in the same gene is a rare finding but it is a well known observation also from other autosomal recessive disorders, e.g. cystic fibrosis or autosomal recessive polycystic kidney disease. This means that at least two mutations are localised on the same allele, and this might lead to false-positive or false-negative results in carrier diagnostics.

### *SLC3A1*

As mentioned before, mutations in *SLC3A1* are generally associated with type I cystinuria, but with the dupE5E9 exceptions exist [10]; heterozygote carriers of this mutations show an increased urinary cystine excretion pattern.

**Figure 2:**
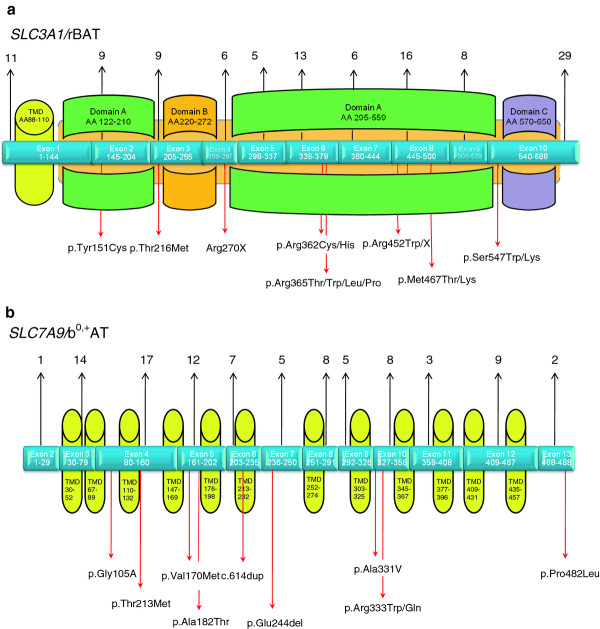
**Genomic structure and putative function of the encoded protein regions of the cystinuria genes: the total number of mutations described so far for each exon is shown above, the localisation of the most frequent mutations is shown below the exon structure.a)***SLC3A1*/rBAT (TMD transmembrane domain; the largest part of the protein consists of an ectodomain (light brown) of three domains A-C, for further details see [4]). **b)***SLC7A9*/b^0,+^AT (TMD transmembrane domain).

Mutations are distributed over the whole gene, affecting all exons and all functional domains (Figure 2). Among the currently known *SLC3A1* mutations p.Met467Thr is generally the most frequent one, accounting for ~30% of the known *SLC3A1* alleles and detectable in nearly all ethnic groups (Table 1).

The amino acid substitution p.Thr216Met in exon 3 is the second frequent variant (~13% of all identified *SLC3A1* alleles), but it shows a preponderance in patients from South-Eastern Europe (for review: [4,25]) and thus reflects the effect of the ethnic origin on the distribution of cystinuria mutations.

For some variants an ethnic influence can be observed: it is mostly obvious for the mutation p.Arg270X which has been identified in 73% of Ashkenazi Jewish patients and accounts for 11% of the known *SLC3A1* mutations in North America (Table 1). The mutation p.Tyr151Cys has mainly been observed in Northern Europe [34]. The duplication dupE5E9 might have occurred from a German founder, however data are limited as tests aiming on larger genomic imbalances were not routinely applied for cystinuria diagnostics in the past.

### *SLC7A9*

The functional consequences of *SLC7A9* mutations are generally broader than that in *SLC3A1*. For many *SLC7A9* mutations, an autosomal-dominant influence can be observed in respect to the urinary amino acid patterns, but the penetrance is incomplete in respect to stone fomation. As a result, a genotype-phenotype correlation is merely possible and a broad intrafamilial biochemical and clinical spectrum for the same mutation can be observed.

Similar to *SLC3A1*, mutations in *SLC7A9* can be detected in all exons of the gene (Figure 1) but there are hot spots for mutations in exons 3, 4, 5 and 6 (Table 1). Among the *SLC7A9* mutations, the amino acid substitution p.Gly105Arg accounts for approximately 20% of identified alleles and is present in nearly all ethnic groups.

The second frequent mutation p.Arg333Trp is also detectable worldwide, whereas other variants are often rare. Interestingly, three mutations show a strong ethnic association: p.Val170Met is restricted to Libyan Jews where it accounts for all patients; p.Pro482Leu affects >84% of the identified *SLC7A9* alleles in the Japanese cystinuria cohort [42]. The duplication c.614dupA is preponderant in Spanish patients, accounting for 29% of *SLC7A9* alleles and probably originating from a founder in Asturias (for review: [4]).

In the group of rare *SLC7A9* mutation, the variant p.Thr123Met should be mentioned separately as it illustrates the broad physiological and clinical phenotype of *SLC7A9* mutations. The amino acid excretion patterns can range from nearly normal to isolated cystinuria to hyperexcretion of cystine and the dibasic amino acids in carriers of p.Thr123Met [10,43].

## The hypotonia-cystinuria syndrome and 2p21 microdeletions

Larger deletions affecting *SLC3A1* might result in the so-called hypotonia-cystinuria syndrome (HCS; OMIM 606407). This autosomal recessive congenital disorder is associated with deletions of at least the *SLC3A1* and *PREPL* genes on chromosome 2p21. The main clinical features include a generalised hypotonia at birth, failure to thrive, growth retardation and cystinuria. Meanwhile 13 patients with HCS have been reported, all were homozygous for deletions in 2p21 [44,45]. So far, five different HCS deletions have been identfied, two of them (deletions “A” and “B”) were found to be globally distributed. Despite their different sizes ranging from ~38 to ~127 kb, they all affect (at least in parts) the *SLC3A1* and *PREPL* genes resulting in functionally homozygous deletions of both genes. A further patient with cystinuria as the only clinical feature was detected by screening cystinuria patients for *SLC3A1* mutations; the patient was compound heterozygous for a *SLC3A1/PREPL* deletion and a deletion in *SLC3A1* affecting exons 1 to 7 [40]. In contrast to the association of *SLC3A1* mutations with cystinuria, variants restricted to *PREPL* causing an “isolated hypotonia” phenotype have not yet been reported. Nevertheless, it has been postulated that the phenotype in HCS patients can be attributed to the lack of *PREPL*, a putative serine oligopeptidase with a currently unknown physiological function [44].

A second microdeletion syndrome also affecting 2p21 but larger in size (~179 kb) is referred to as 2p21 deletion syndrome [46]. This deletion has been detected in a large Bedouin pedigree. Homozygous deletion carriers in this family showed HCS and additional features including neonatal seizures and a severe global retardation, indicating that the loss of a third gene in 2p21, *PPM1B*, also contributes to the clinical spectrum in this family. Meanwhile, additional patients with an intermediate phenotype between HCS and the 2p21 microdeletion syndrome and deletions intermediate in size have been described [47].

## Further cystinuria candidate genes

Considering the observation that in many studies the detection rates for mutations in *SLC3A1* or *SLC7A9* do no reach 100% and due to the complex nature of renal amino acid transport, the role of further genes and modifying factors in the etiology of cystinuria have been postulated. However, linkage analyses in cystinuria families did not indicate the existence of more than two cystinuria loci, 2p21 (*SLC3A1*) and 19q13 (*SLC7A9*), therefore the localisation of further genes encoding amino acid transporter subunits within this region was conceivable.

One candidate in 19q13 was *SLC7A10* (ASC-1) which shows high homology with *SLC7A9* but several studies excluded pathogenic mutations in this gene [31,48-50]. The neutral amino acid transporter system ATB(0) (*SLC1A5*) is also localised in 19q13, but mutation analysis in cystinuria families with possible linkage to this region did not provide evidence for a contribution of *SLC1A5* mutations to the clinical course [32].

The lack of mutations on single alleles can also be explained by the physiological dominance of some *SLC7A9* alleles, in these cases a second mutation is not needed to influence the phenotype. Furthermore, it has been suggested that at first glance apathogenic single nucleotide polymorphisms (SNPs) might predispose a cystinuria phenotype in case of co-occurrence with other mutations [28,51] and might serve as modifiers.

## Genetic testing strategies for cystinuria

The developing of genetic tests for cystinuria reflects the rapid technological evolution in the last two decades. Molecular genetic testing was firstly established after the identification of the rBAT cDNA and first mutations in 1994. At that time the genomic structure of *SLC3A1* was not known and single PCRs with subsequent restriction digests were established for specific mutations. With the characterisation of the genomic structure of both genes, unspecific screening methods like SSCP (single strand conformation polymorphism analysis) were applied to screen large cohorts of patients. However, these tests were limited due to their sensitivity and reproducibility, therefore Sanger sequencing became the gold standard for molecular genetic analysis in cystinuria. To circumvent the problem that large rearrangements not covered by conventional sequencing approaches might escape detection, several quantitative assays, i.e. quantitative PCR and *multiplex ligation probe-dependent amplificiation* (qPCR and MLPA), have been implemented in diagnostic algorithms.

Based on the growing knowledge on the genetic and biochemical basis of cystinuria, the following aspects should be considered prior to molecular genetic testing:

- biochemical and clinical data might influence the diagnostic algorithm: in the majority of cases, patients are referred with the clinical diagnosis of urolithiasis. In this situation the careful family history and urinary amino acid excretion patterns from the parents might help to decide which gene should be tested first: in case of sporadic occurrence and normal parental urinary amino acid excretion, *SLC3A1* should be tested, in case of a positive family history and parental hyperaminoaciduria, *SLC7A9* testing should be prioritized. However consanguinity might mimic an autosomal recessive inheritance and mutations in both genes show a broad observable range of urinary cystine excretion. Furthermore, the comprehensive data are often difficult to obtain or information of family members are not available.

- ethnic origin: for specific populations (e.g. Japan, Sweden, South-East Europe, Spain) a targeted algorithm can be applied as these groups show specific mutations.

*-*testing procedure: Frequent or population-specific mutations might be tested by targeted genotyping approaches (e.g. restriction assays). In case of sequencing of the coding regions and the intron/exon boundaries, one might consider to start with the “hot spot” exons harbouring multiple mutations and frequent variants (e.g. exon 8 of *SLC3A1*, exon 4 of *SLC7A9*). In case none of these frequent mutations is detectable, the total coding sequences of both genes have to be sequenced. After exclusion of exonic mutations, quantitative tests should follow as intragenic duplications/deletions significantly contribute to the spectrum of mutations in cystinuria [24]. In case of unusual phenotypes, molecular karyotyping with DNA microarrays is an appropriate tool to identify large genomic imbalances as reported for the HCS [44]. This step-wise analysis can be stopped after identification of two pathogenic variants explaining the clinical course of the patient, although the presence of a third mutation [27,31] can not be excluded.

## Cystinuria and genetic counselling

In contrast to other disorders characterised by urolithiasis, cystinuria is solely caused by genomic mutations. Nevertheless, the interpretation of the molecular results and genetic counselling in cystinuria families is often complicated by the difficulty to differentiate between patients and heterozygote carriers of mutations with ambiguous clinical significance. In these situations, a prediction of the clinical course or a targeted therapeutic regime is often asked by the patients and their families. However, an unambiguous prognosis is merely possible due to the broad clinical variability even in carriers of the same mutation and the influence of so far unknown modifying endo- and exogenous factors. Nevertheless, a more-directed counselling has become possible based on the molecular classification suggested by DelloStrologo et al. [11]:

- **AA** (homozygosity for one or compound heterozygosity for two *SLC3A1* mutations) is mainly consistent with an autosomal recessive inheritance of cystinuria. The parents are obligate carriers but with a normal renal cystine excretion, a recurrence risk of 25% can be delineated for children to be affected by cystinuria. However, there is evidence at least the mutation dupE5E9 is associated with non-type I cystinuria, i.e. carriers show an increased cystine excretion [10]. Heterozygote carriers of this variant should be tested for urinary cystine excretion.

- **A?** (heterozygosity for a *SLC3A1* mutation, a second mutation could not be identified): probably a second mutation in one of the two genes is present but not detectable by the applied methods. However, in the case of clinical diagnosis of cystinuria, the identification of only one mutation in *SLC3A1* should be sufficient for confirmation. To further estimate the recurrence risk in these families the urinary excretion patterns and the cystinuria genotype of the parents should be determined. In case of a normal cystine excretion in that parent not contributing the *SLC3A1* mutation compound heterozygosity for the identified and an unknown *SLC3A1* mutation can be delineated for the patient. If the parent not carrying the detected *SLC3A1* mutation shows an increased cystine excretion, a *SLC7A9* mutation can be assumed. In that case the clinical prediction for the offspring expected to be heterozygous for the (unknown) *SLC7A9* mutations is difficult because of the broad range of biochemical penetrance of mutations in this gene.

- **BB** (homozygosity for one or compound heterozygosity for two *SLC7A9* mutations) is consistent with cystinuria. The recurrence risk for sibs is at least 25%, but due to the possible dominant nature of *SLC7A9* mutation and the broad ranges of urinary amino acid excretion even in the same family the risk to develop cystine stones is higher. Here the biochemical analysis of the urine of heterozygote mutation carriers and the knowledge of the pathogenic nature of the mutation might help to further delineate the risk to develop stones.

- B? (heterozygosity for a *SLC7A9* mutation, a second mutation could not be identified): this finding is the most difficult one to interpret: as discussed for the BB genotype, the same mutation might behave recessive in one generation and dominant in another in the same family. Thus the identification of just one *SLC7A9* mutation might be compatible with an extremely increased cystine excretion, the prognosis in newborns or children is merely possible.

- **AB** (mixed heterozygosity of a *SLC3A1* and a *SLC7A9* mutation): this finding explains the clinical phenotype, but as discussed for the A?, BB and the B? genotypes, the probable dominant effect of a *SLC7A9* mutation has to be bare in mind in genetic counselling.

- **AAA/AAB/ABB** (three *SLC3A1/SLC7A9* mutations in one patient): this rare finding explains the phenotype, but the parents should be checked for the mutations to identify the chromosome harbouring two mutations and thereby to avoid false-negative carrier testing results in the further family.

In clinical practice, the molecular genetic testing results only scarcely influence the prognosis and therapy of cystinuria as causative therapies do currently not exist. Indeed, more than 95% of all carriers of two *SLC3A1* or *SLC7A9* mutations (genotypes AA, BB, AB) will develop kidney stones in their live but the age of kidney stone formation is difficult to predict and shows a broad intrafamilial variability. Furthermore, the urinary excretion pattern in heterozygote *SLC7A9* mutation carriers is extremely variable and does therefore hardly allow a prediction of the clinical course whereas the majority of *SLC3A1* heterozygotes do not exhibit a biochemical phenotype (for review: [12]). As far as the comprehensive knowledge on the pathophysiology of cystinuria can not be applied in a causative therapy, the biochemical determination of the urinary cystine excretion pattern remains the basic tool for prognosis and therapeutic management of cystinuria.

## Competing interests

The authors declare that they have no competing interests.

## Authors’ contributions

TE wrote the draft of the manuscript. All authors discussed, read and approved the manuscript.
